# Host and microbiota derived extracellular vesicles: Crucial players in iron homeostasis

**DOI:** 10.3389/fmed.2022.985141

**Published:** 2022-10-13

**Authors:** Yasmeen Daou, Marion Falabrègue, Charareh Pourzand, Carole Peyssonnaux, Marvin Edeas

**Affiliations:** ^1^International Society of Microbiota, Tokyo, Japan; ^2^INSERM, CNRS, Institut Cochin, Université de Paris, Paris, France; ^3^Laboratory of Excellence GR-Ex, Paris, France; ^4^Department of Life Sciences, University of Bath, Bath, United Kingdom; ^5^Medicines Development, Centre for Therapeutic Innovation, University of Bath, Bath, United Kingdom

**Keywords:** iron metabolism, extracellular vesicles, gut microbiota, ferritin, transferrin receptors, hepcidin

## Abstract

Iron is a double-edged sword. It is vital for all that’s living, yet its deficiency or overload can be fatal. In humans, iron homeostasis is tightly regulated at both cellular and systemic levels. Extracellular vesicles (EVs), now known as major players in cellular communication, potentially play an important role in regulating iron metabolism. The gut microbiota was also recently reported to impact the iron metabolism process and indirectly participate in regulating iron homeostasis, yet there is no proof of whether or not microbiota-derived EVs interfere in this relationship. In this review, we discuss the implication of EVs on iron metabolism and homeostasis. We elaborate on the blooming role of gut microbiota in iron homeostasis while focusing on the possible EVs contribution. We conclude that EVs are extensively involved in the complex iron metabolism process; they carry ferritin and express transferrin receptors. Bone marrow-derived EVs even induce hepcidin expression in β-thalassemia. The gut microbiota, in turn, affects iron homeostasis on the level of iron absorption and possibly macrophage iron recycling, with still no proof of the interference of EVs. This review is the first step toward understanding the multiplex iron metabolism process. Targeting extracellular vesicles and gut microbiota-derived extracellular vesicles will be a huge challenge to treat many diseases related to iron metabolism alteration.

## Introduction

Iron is a vital trace element, essential for several fundamental processes: globin synthesis and erythropoiesis (1), energy production (2), DNA synthesis and repair (3), and immune function (4). Its mode of action lies in its ability to reversibly gain or lose a single electron to participate in oxidation-reduction reactions, which also catalyze the generation of reactive oxygen species (ROS) (5). Hence, in spite of its vital role iron overload is toxic due to its ability to generate ROS and trigger cell death (6). Excess of free reactive iron leads to several types of cell death, including ferroptosis, the oxidative cell death prompted by the accumulation of iron-mediated lipid peroxidation (6).

No wonder, iron metabolism is a tightly regulated process. The regulation of iron metabolism occurs at a cellular level via transcriptional and post-transcriptional regulation of iron genes. At the systemic level, iron metabolism is regulated via the hepatic hormone hepcidin, which regulates iron absorption, plasma concentrations, and tissue distribution (7). The human microbiome, in turn, was recently identified as an effector in the iron metabolism regulation process, considering the fact that the microbiota also requires iron to maintain symbiosis. The composition of the gut microbiota is affected by iron availability, and microbiota-derived metabolites were reported to impact both iron absorption (enterocyte) and iron recycling (macrophage).

The iron metabolism process and its regulation are highly complex; up till now, there remain a lot of missing links. Ferritin, a main cargo of blood iron, and transferrin receptors, involved in the cellular membrane transportation of iron, have been identified in vesicular locations (8–10). Also, bone marrow-derived extracellular vesicles (EVs) in β thalassemia patients were reported to affect hepcidin production (11). However, no paper has yet discussed the possible role of EVs in iron homeostasis. Considering the blooming significance of extracellular EVs in cell–cell communication, delivering cargo, and modulating the physiological condition (12); we suggest that EVs might play a role in transporting iron regulators thus maintaining iron homeostasis.

Here we will discuss the critical process of iron metabolism and the systems of its regulation, while focusing on the potential interference of extracellular vesicles. We will also discuss the role of gut microbiota and its metabolites in iron homeostasis, and consider the potential involvement of microbiota-derived extracellular vesicles in this relationship.

## Iron metabolism

Iron metabolism consists of iron absorption, use, storage, and transfer. Absorbable dietary iron can be in the form of heme and non-heme iron (13). Heme iron contributes to 10–15% of the absorbed dietary iron. It is more absorbable by the body (15–35%) as compared to non-heme iron (2–20%) (14). Even though heme iron absorption surpasses that of non-heme iron, its absorption mechanisms remain ambiguous. Non-heme iron is absorbed at the level of the duodenum and proximal jejunum, by enterocytes divalent metal-ion transporter 1 (DMT1), after being reduced to ferrous iron (Fe^2+^) via duodenal cytochrome b (Dcytb) (15, 16). If not needed inside the cell, ferrous iron, is either stored in the form of ferritin or transferred to circulating transferrin (TF) via the iron exporter ferroportin (FPN), after being oxidized to ferric iron (Fe^3+^) by hephaestin (17). Transferrin bound iron is delivered to sites of utilization, where it binds to cell surface transferrin receptor 1 (TFR1) and endocytose into the cell (18); it then enters the cytoplasm via DMT1 in the endosomal membrane, after being reduced to (Fe^2+^) under the action of six-transmembrane epithelial antigen of prostate 3 (STEAP3) (19). This iron can be used for metabolic functions, or stored within cytosolic ferritin- iron in the ferric form associated with hydroxide and phosphate anion (20).

Merely 1–2 mg of dietary iron are absorbed in the gut daily; most iron is recycled upon phagocytosis of erythrocytes by macrophages (21). Senescent/damaged erythrocytes are phagocytosed by macrophages. Macrophages recover iron from heme via heme oxygenase 1 (HMOX1) for utilization, conservation or recycling depending on the body needs (22).

## Iron homeostasis

The iron metabolism process is highly critical, considering the danger of excess free reactive iron. It is supposed to be tightly regulated. Iron homeostasis is essential to maintain normal physiology. To achieve homeostasis, iron regulation takes place at both cellular and systemic levels. At the cellular level, iron homeostasis involves mechanisms that balance iron uptake with intracellular iron storage and utilization (23). Systemic iron homeostasis embodies the mechanisms that synchronize dietary iron absorption and iron concentration in plasma and the extracellular milieu (24).

### Iron homeostasis at the cellular level

At the cellular level, iron metabolism regulation is based on transcriptional and post-transcriptional regulation of iron genes. Post transcriptional regulation is facilitated by the binding of iron regulatory proteins (IRPs) to iron-responsive elements (IREs) of their mRNA untranslated regions (25). In case of iron-deficiency, IRP1 and IRP2 bind to the IREs in TFR1 mRNA and stabilize it, thus increasing iron uptake. They also bind to the 5′-UTR of the mRNAs that encode ferroportin and ferritin to suppress their translation, thus blocking iron export and storage (26). Conversely in iron-replete cells, iron can bind IRPs and induce their conformational change. Notably, IRP1 assembles an aconitase-type 4Fe-4s and this assembly alters its conformation (27, 28). The latter conformational changes weaken the IRPs IRE-binding ability, thus leading to (a) destabilization and degradation of TFR1 mRNA and (b) facilitated translation of the target 5′-UTR mRNA encoding ferroportin and ferritin (29). Micro RNAs (miRNAs) also count as significant posttranscriptional regulators of gene expression. They can regulate cellular iron homeostasis by influencing iron absorption, transport, storage, and utilization. For example, miR-Let-7d was reported to target the DMT1-non-IRE isoform (30) and miR-320 was found to post-transcriptionally control TfR1 expression (31). The mRNA encoding FPN in turn was shown to be targeted by miR-485-3p (32). Furthermore, the storage of iron as ferritin is downregulated by miR-200b (33).

Transcriptional regulation is dependent on hypoxia inducible factors (HIFs). Hypoxia inducible factor -2α (HIF-2α) plays an important physiological role in transcriptional regulation of iron homeostasis. HIF-2α regulates iron absorption notably by activating the expression of DcytB and DMT1 proteins during iron deficiency or ineffective erythropoiesis to increase iron uptake (34, 35). In fact, *HIF-2*α mRNA 5′-UTR contains an IRE that binds both IRP1 and IRP2 (36, 37) for iron-dependent regulation of the transcript. By regulating HIF-2α mRNA, IRP1 amends the erythropoietic response to hypoxia. This IRP1-HIF-2α axis synchronizes both iron and oxygen sensing with erythropoiesis and iron absorption (38). Furthermore, HIF regulates hepcidin, the orchestrator of systemic iron homeostasis, via erythropoietin-induced erythropoiesis. HIF can suppress the hepcidin gene *Hamp1* indirectly through erythropoietin-induced erythropoiesis (39, 40).

### Iron homeostasis at the systemic level

Systemic iron homeostasis is regulated via hepcidin, a hormone that is primarily secreted by hepatocytes. Iron and inflammatory cytokines induce hepcidin expression, while iron deficiency, erythropoiesis, and anemia/hypoxia downregulate it (17). High circulating iron levels upregulate hepcidin expression by hepatocytes, through the BMP/SMAD pathway (41). The binding of transferrin to transferrin receptor 2 (TFR2), to which it has less affinity than TFR 1, in case of high plasma iron concentration, has been anticipated to affect hepcidin expression. The binding of diferric transferrin to TFR2 induces overexpression of hepcidin in hepatocytes and reduced erythropoietin responsiveness in erythroid cells (42), where TFR2 binds erythropoietin receptors (43). Inflammatory cytokines upregulate hepcidin gene expression through the Janus kinase/signal transducers and activators of transcription (JAK/STAT); for example, IL-6, increases hepcidin expression via activating the IL-6R-JAK2-STAT3 pathway (44). When activated, hepcidin combines FPN1, internalizes it, and degrades it in the lysosome (45), thereby reducing iron absorption by duodenal cells and iron recycling by macrophages.

## Extracellular vesicles: Key players in iron homeostasis?

**FIGURE 1:**
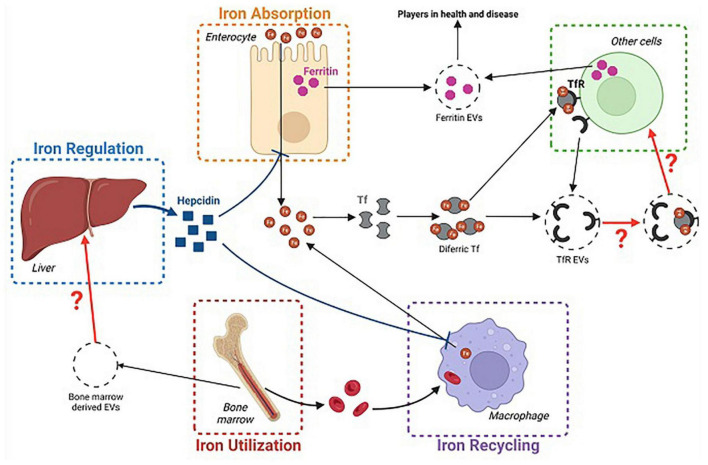
Schematic representation of the potential implication of extracellular vesicles (EVs) in iron homeostasis. This figure illustrates the possible involvement of (1) Transferrin carrying EVs (TFR EVs) in iron delivery to host cells, (2) Ferritin EVs in mediating disease symptoms, and (3) Bone marrow-derived EVs in affecting hepcidin release. ©Created with BioRender.com.

Extracellular vesicles are membrane-bound vesicles secreted by cells into the extracellular space. They constitute microvesicles, exosomes, and apoptotic bodies- released by dying cells; they vary in size with microvesicles being the smallest (less than 100 nm–1 μm), followed by exosomes and apoptotic bodies, respectively (46). They function by facilitating the intercellular exchange of proteins, lipids, and genetic material, thus facilitating intercellular signaling/communication (47). While it is known that iron metabolism requires a lot of cell communication, the exact role of EVs in either iron metabolism or the maintenance of iron homeostasis remains to be elucidated. The putative role of EVs in iron homeostasis is summarized in Figure 1.

### Ferritin extracellular vesicles and their fate

Iron is stored in the cells in the form of ferritin, as mentioned before. The synthesis and degradation of ferritin are both orchestrated by cellular iron status. Under low iron levels, ferritin synthesis decreases via translational repression (48), and ferritin lysosomal degradation, mediated by the selective autophagy receptor NCOA4, increases. The degradation of the ferritin to free the iron stored is a selective macro-autophagy mechanism called ferritinophagy and is mediated by NCOA4 (49). On the other hand, high iron levels decrease the entry of ferritin into the lysosome (50). Ferritin can be found in the cytoplasm (51), nucleus (52), and mitochondria (53). However, it has also been observed in vesicular locations.

Some researches succeeded to report the presence of ferritin in human urinary exosomes, via a large-scale proteomic analysis (9). However, there is a gap in our knowledge underlying ferritin intracellular trafficking and secretion. It was thought to be secreted through the Endoplasmic Reticulum–Golgi route (54), but Cohen et al. showed that ferritin is secreted primarily by macrophages through lysosomal pathway, by secretory lysosomes (55). Truman-Rosentsvit et al. provided evidence on the secretion of ferritin via the multivesicular body–exosome pathway (56). Yanatori et al. studied the mechanism of secretion of ferritin in extracellular vesicles (57). They discovered that CD63, which plays a role in EVs secretion (58), is post-transcriptionally regulated by iron via the IRE-IRP system, the system that induces ferritin under iron upsurge (59). Thus, increased levels of iron induce CD63 expression. Upon loading iron, intracellular ferritin is transported via nuclear receptor coactivator 4 (NCOA4)/ferritin vesicles to CD63^+^ EVs that are secreted (57).

Exosomes play a role in iron homeostasis after all, but the destination of ferritin carried by the exosomes remains to be explored. Is it transferred to hepatocytes, the major sites for iron storage? Can those exosomes fuse with the free mitochondria and deliver ferritin to them? Can the ferritin stored in mitochondria be released via mitochondria-derived vesicles (MDVs) too? Vasam et al. had reported that MDVs revealed high levels of mitochondrial iron–sulfur clusters biogenesis proteins, that are responsible for the biogenesis of iron-containing cofactors, and iron-binding capable proteins. They suggested that MDVs can serve as a potential source of biomarkers for mitochondrial stress (60).

The serum ferritin level mirrors the body’s iron stock; it is considered a hematologic index for iron-associated diseases (61). Whether this serum ferritin is contained in EVs or possibly released from ferritin carrying EVs is ambiguous. Excessively elevated levels of both ferritin (62, 63) and EVs (64) have long been noted in the circulation of iron overload β-thalassemia patients. Recently, Atipimonpat et al. reported the presence of high levels of ferritin-bearing exosomes in the plasma of β-thalassemia patients (65). They explained that those high levels of circulating red blood cells and activated platelets derived EVs, especially ferritin-carrying exosomes, can speed up the proliferation of H9C2 cardiac cells leading to cell hyperplasia, progression of cardiac hypertrophy, and eventually heart failure. What remains to be clarified is whether iron overload can induce or increase the ferritin-loaded exosomes and if ferritin-loaded exosomes can contribute to disease symptoms.

Strzyz in turn reported ferritin exosomes to induce ferroptosis resistance (66). In this regard, exosomes provide a route for ejecting iron out of ferroptotic cells, thus protecting them from ferroptosis. Accordingly, Mukherjee et al. reported that disrupting the EV release or ferritin heavy chain expression in oligodendrocytes resulted in neuronal loss and oxidative damage in mice (67). In case of cancer, carcinoma cells may use this iron export pathway involving multivesicular body/exosome trafficking of iron out of the cell to avoid ferroptotic death (68). Interestingly, a study conducted by Ito et al. showed that macrophages that engulf asbestos produce ferroptosis-dependent extracellular vesicles that contain ferritin and transport it to mesothelial cells, thereby contributing to mesothelial carcinogenesis by loading ferritin (69). Alterations in EV levels during therapy have been reported in Glioblastoma patients; interestingly the EV protein signature showed common iron metabolism proteins and disappeared post-surgical resection (70). We can speculate that ferritin extracellular vesicles can halt ferroptosis in health and in disease. They show potential in becoming biomarkers for disease diagnosis, notably cancer.

### Transferrin receptor carrying extracellular vesicles

Transferrin receptor expression was also detected in exosomes. The presence of Glyceraldehyde-3-phosphate dehydrogenase (GAPDH), which has been characterized as a transferrin receptor (71, 72), in exosomes from different cell lines was confirmed by Malhotra et al. along with its ability to bind transferrin (10). They also reported that iron preloaded exosomes delivered more iron into various cells, thus raising an interesting chance of exosomes playing a role in the delivery of iron and iron homeostasis. Interestingly a recent study conducted by Dar et al. demonstrated that GAPDH also induces clustering of EVs *in vitro* and *in vivo* (73). Further, Mattera et al. identified the expression of TFR1 in extracellular vesicles derived from human and mouse plasma, rat oligodendroglioma cells, mouse neuroblastoma cells, and rat astrocytes (8). However, no research has been conducted on the presence of TFR2, that induces hepcidin expression upon binding diferric transferrin, in hepatocytes and erythroblasts derived EVs.

### Extracellular vesicle-mediated hepcidin modulation

Hepcidin, the key iron regulator, is produced primarily by hepatocytes neighboring the portal veins and Kupffer cells (7), macrophages (74), adipocytes (75), and dendritic cells (76). Also, several studies have reported local synthesis of hepcidin by multiple other tissues, notably in disease, like the and the lungs (77), kidney (78), stomach (79), adipose tissue (75), brain (80), heart (81), and even the skin (82). The organ that produces the most hepcidin after the liver is the heart (83). As mentioned before, under active erythropoiesis, hepcidin production is somewhat inhibited via erythroferrone, an erythroid factor produced by the erythroblasts, that suppresses the BMP/SMAD pathway in the liver (84). β-Thalassemia patients are known to have ineffective erythropoiesis and iron overload. This ineffective erythropoiesis suppresses hepcidin leading to iron overload (85). Ruiz Martinez et al. hypothesized that bone-marrow derived exosomes modulate hepcidin expression and regulate iron metabolism (11); they investigated the link between exosomes and hepcidin regulation in β-thalassemia. They were able to demonstrate that those exosomes boost hepcidin expression by increasing SMAD1/5/8 signaling. Increased hepcidin, in response to exosomes, will possibly influence several signaling pathways by an autocrine mechanism. Exosomes compensated for suppressed hepcidin in the exosome-depleted serum of β-thalassemic samples. Proteomic analysis of β-thalassemic patients’ bone marrow derived exosomes can help us better understand their role in hepcidin regulation. On the other hand, EVs derived from the plasma of β-thalassemic patients showed dysregulation of certain miRNAs involved in oxidative stress, erythropoiesis, and apoptosis- in particular overexpressed miR-144-3p. They were reported to induce apoptosis in endothelial, pancreatic, and hepatic cells, possibly contributing to the organ damage in β-thalassemia (86). Thus, studying EVs derived from β-thalassemic patients not only can help to understand the implication of EVs in iron metabolism regulation, but they can also help to comprehend the health complication associated with β-thalassemia. They can also serve as biomarkers for β-thalassemia severity.

## Gut microbiota: Modulation of iron homeostasis

Almost all living organisms require iron in order to survive. A deficiency or excess in iron is dangerous hence iron homeostasis is firmly regulated. Iron acquisition takes place at the level of the small intestine (87). The small intestine is colonized by symbiotic microorganisms, called the “gut microbiota,” that share a mutually beneficial relationship with the host (88, 89). The gut microbiota relies on the host for nutrients and survival, while it plays an indirect role in regulating complex endocrine networks. Gut microbiota compete with the host to acquire iron, for survival. Studies have reported how iron deficiency/repletion in rats (90) or genetic modification of iron metabolism in mice (91) affect the gut microbiota composition and metabolic activity. The fecal microbiota has even been proposed as a non-invasive biomarker for tissue iron accumulation prediction in intestine epithelial cells and liver (92).

The gut microbiota must in turn also impact the iron absorption process and play an indirect role in regulating iron homeostasis. Most studies, however, focus on the strategies used by microbiota to acquire iron, studies on the possible role of microbiota in regulating iron homeostasis are scarce. Deschemin et al. were the first to investigate the impact of microbiota on host iron sensing (93). They demonstrated that gut microbes induce a specific iron-related protein signature and revealed a new feature of the microbiota – intestinal epithelium crosstalk. Further, Das et al., in their turn, then studied the impact of gut microbiota on iron intestinal absorption, and discussed its unforeseen role in regulating host iron homeostasis (94). They identified that the host iron-sensing mechanism is connected to the gut microbiome and regulated by it. They reported that, intestinal iron deficiency leads to the positive selection of *Lactobacillus* species that produce reuterin and 1,3 diaminopropane (DAP). Those microbiota metabolites (a) suppress iron absorption, by inhibiting the transcription factor HIF-2α that targets the expression of key iron transporters (34, 35, 95), and (b) induce ferritin expression to eventually lead to its degradation by the host in due to the iron overload caused - in order to maintain homeostasis (34); thereby indirectly preventing tissue iron accumulation (94).

**FIGURE 2:**
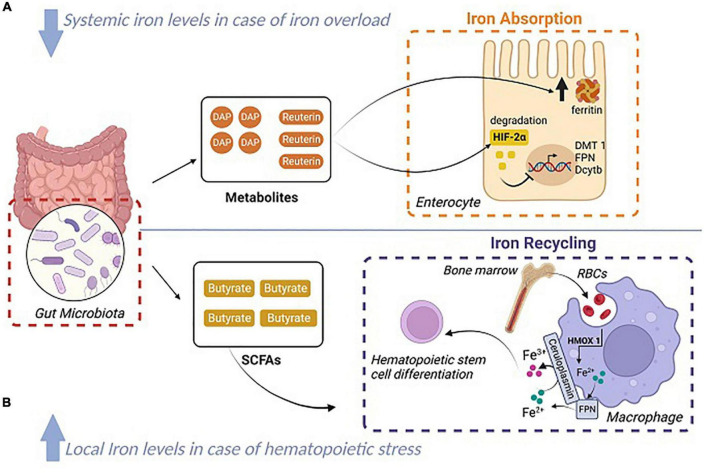
Summary of the achieved relationship between the gut microbiota and systemic/local iron levels. **(A)** The gut microbiota releases 1,3 diaminopropane (DAP) and reuterin in case of iron overload and decreases systemic iron levels. DAP and reuterin induce ferritin expression, and cause the degradation of HIF-2α consequently blocking the expression of the iron transporters (Dcytb, DMT1, and FPN). **(B)** Hematopoietic stress leads to the release of butyrate by the gut microbiota. Butyrate decreases local iron levels by promoting iron recycling (erythro-phagocytosis) in order to allow hematopoietic stem cells differentiation. ©Created with BioRender.com.

From another perspective, Zhang et al. also reported that microbiota derived short chain fatty acids (SCFAs), specifically butyrate, can lead to iron distribution, for fueling hematopoietic regeneration, by promoting emergency erythro-phagocytosis by bone marrow macrophages (96). However, it is important to note that microbiota depletion resulted in only reduced local iron levels without affecting systemic iron homeostasis (96). Yet, future studies should further explore a possible involvement of a microbiota-macrophage-iron axis in iron homeostasis. All the current knowledge on the relationship between gut microbiota and host iron levels is summarized in Figure 2.

In reality, we are still in the infancy of understanding the metabolic crosstalk between gut microbiota and the host intestinal epithelium- in terms of iron homeostasis. A lot of studies are required to identify the role of microbiota-derived metabolites in context of iron homeostasis, and to determine whether microbiota commensals can secrete hormones that directly regulate or impact iron metabolism. Additionally, it is important to study the potential of targeting gut microbiota therapeutically via prebiotics, probiotics, or fecal microbiota transplant for iron-related diseases.

### Gut microbiota and hepcidin production: An intriguing question

As explained before, hepcidin is the central iron regulator, it is overexpressed in response to iron overload and inflammation (97); it blocks iron export (98) and degrades the iron exporter ferroportin (45), thereby reducing iron absorption by duodenal cells and iron recycling macrophages. The microbiota produces a wide range of metabolite-derived humoral agents including SCFA, secondary bile acids, and neurotransmitters all of which play important body functions (99). As mentioned above, *Lactobacillus* derived metabolites were lately reported to play an indirect role in regulating iron homeostasis.

The secretion of hepcidin by myeloid cells occurs via the toll-like receptor 4-hepcidin pathway as a host response to bacterial pathogens (100). Layoun and Santos showed that bacterial cell wall lipopolysaccharide induced hepcidin expression in macrophages (101). Microbiota dysbiosis in the inflamed intestine of humans was found to induce the release of hepcidin by conventional dendritic cells, for tissue repair (76). However, one intriguing question that remains to be answered is whether the microbiota can modulate iron homeostasis, directly, by producing hepcidin.

### Gut microbiota derived extracellular vesicles: Players in iron homeostasis?

Iron is essential for the majority of microorganisms. Some bacteria have evolved efficient strategies to acquire iron from the host. The microbiota acquires iron from the host by using host iron compounds (e.g., heme, transferrin, lactoferrin ferritin), producing high-affinity iron chelators called siderophores, and/or uptaking ferrous iron (102). Gram-negative bacteria derived outer membrane vesicles (103) and gram-positive bacteria derived extracellular vesicles (104) are well reported to play significant roles in bacterial survival, material exchange, cell-to-cell communication, and pathophysiology. EVs of some pathogenic bacteria are known to help them acquire iron from the host (105). Additionally, bacteria-derived EVs (*Diaetzia sp.*) were found to allow homologous bacterial species to share iron (106).

Microbiota-derived EVs are involved in inter-kingdom communication with host cells in the gut (107, 108). They were reported to deliver to host cells effector molecules that modulate host signaling pathways and cell processes (108). The probable effects of gut microbiota–derived EVs on metabolic diseases such as obesity and diabetes have been reviewed (109). Knowing that the gut microbiota plays an indirect role in regulating the host’s iron homeostasis, no research has yet addressed the potential role of microbiota-derived EVs in the regulation of iron homeostasis.

Bacteria can store iron in the form of bacterial ferritin, heme-containing bacterioferritin, and DNA binding dodecameric ferritin (110). The existence of bacterial ferritin, the prototype of ferritin that possesses a classical ferritin H-chain with ferroxidase activity (111), in microbiota-derived EVs might suggest their interference in host iron homeostasis. In fact, Zakharzhevskaya et al. were able to identify non-heme ferritin with special oxidoreductase activity in toxigenic *Bacteroides fragilis* (112). Further research is required to test for the presence of bacterial ferritin in microbiota-derived EVs and unravel the possibility of the association of bacterial ferritin-carrying vesicles with host iron homeostasis.

**FIGURE 3:**
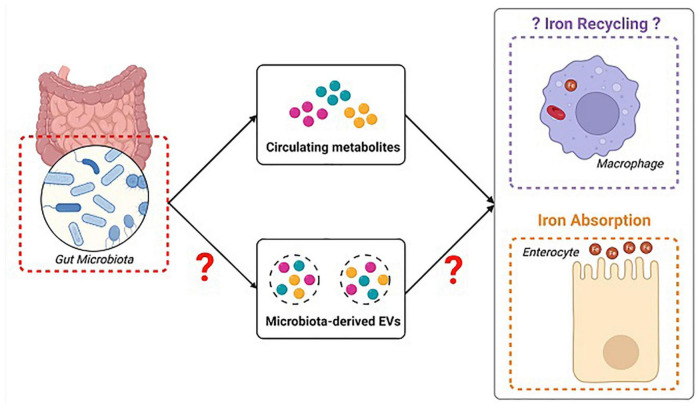
Schematic representation of the putative role of metabolite carrying microbiota-derived extracellular vesicles (EVs) in modulating iron homeostasis. Gut microbiota metabolites mainly interfere in iron homeostasis by acting on iron absorption at the level of the gut. They may possibly interfere with heme recycling at the level of the macrophages. We propose that those metabolites can be released in EVs. ©Created with BioRender.com.

The presence of iron modulating microbial metabolites in microbiota-derived EVs should also be addressed. The potential implication of metabolite-carrying microbiota-derived EVs in the microbiota host iron regulatory mechanisms is depicted in Figure 3. As reported above, the gut microbiota plays a role in regulating the host’s iron homeostasis via *Lactobacillus* derived metabolites reuterin and DAP (94). Moreover, proteomic analysis of *L. reuteri* derived EVs revealed the presence of different functional and structural proteins signifying the possible involvement of *L. reuteri* EVs in metabolism, transport, and signaling (113). Further studies ought to specifically test for the presence of the bacteriocins reuterin and DAP in *L. reuteri*-derived EVs, taking into consideration that species like *Lactobacillus acidophilus* can carry bacteriocin peptides via EVs in order to deliver them to opportunistic pathogens (114).

The presence of SCFAs specifically butyrate in EVs should also be tested to assess the prospective involvement of bacteria-derived EVs in modulating the iron recycling process. *Streptococcus pneumoniae* derived EVs for example were enriched in short chain saturated fatty acids (115).

Thus, the characterization of bioactive molecules and different types of cargo of gut microbiota– and probiotic-derived EVs is required. Once this is achieved, we can begin to explore the potential role of microbiota- derived EVs in regulating the host iron homeostasis. However, it is important to note that there are current limitations to characterize those EVs and their cargo due to the similarity between bacterial and mammalian EVs (107).

## Conclusion

Maintaining iron homeostasis is a challenge reflected by the complexity of iron metabolism. EVs seem to be indispensable in mediating this process. They appear to be key players in iron homeostasis, ferritin is released via exosomes under high iron levels, transferrin receptors are also expressed in exosomes. The question of the destination of ferritin carrying EVs has yet to be answered. Is ferritin transferred via EVs to be stored in hepatocytes? Do they serve in delivering ferritin to free mitochondria? Do mitochondria export iron via MDVs? On another note, EVs should be tested for any ability to deliver iron to distal sites especially to the brain knowing that they can cross the blood brain barrier (116).

Extracellular vesicles are involved in iron regulation in health and disease. Bone marrow-derived EVs can regulate iron homeostasis by compensating for low hepcidin in β-thalassemic patients. However, plasma-derived EVs were also reported to contribute to organ dysfunction and complications of β-thalassemia. A better understanding of EVs composition and cargo might help identify new mechanisms underlying iron-overload diseases such as β-thalassemia or even treat them. Ferritin-containing EVs were reported to suppress ferroptosis in health, but also promote cell survival and proliferation in cancer. This opens doors for better diagnostics and therapeutic options in the future; what if cancer can be diagnosed by identifying ferritin-containing EVs? What if it can be treated by targeting those EVs and modulating iron levels?

Achieving iron homeostasis is also linked to the gut microbiota, the endocrine organ that keeps surprising us with its importance day by day. Gut microbiota metabolites can participate in achieving iron homeostasis by acting on intestinal iron absorption, and can impact local iron levels by affecting bone marrow macrophage erythro-phagocytosis. Knowing that the gut microbiota secretes a wide range of metabolite-derived humoral agents, can it regulate iron metabolism by directly releasing hepcidin? We are still in the infancy of understanding the many roles of gut microbiota in achieving/maintaining iron homeostasis. Later studies should address the effect of gut dysbiosis on iron homeostasis. They should also investigate where microbiota-derived EVs stand in this gut microbiota - iron homeostasis relationship. The future of medicine lies in the microbiota (117). Microbiota derived EVs might be key in future therapies for iron related diseases. Targeting host and gut microbiota-derived EVs will be a huge challenge to prevent and treat many diseases related to the alteration of iron homeostasis and metabolism.

## Author contributions

ME: conception. ME, MF, CPo, CPe, and YD: writing, interpretation, and critical revision of the manuscript. All authors have read and agreed to the published version of the manuscript.

## Abbreviations

EVs: extracellular vesicles
ROS: reactive oxygen species
DMT1: divalent metal-ion transporter 1
Dcytb: duodenal cytochrome b
TF: transferrin
FPN: ferroportin
STEAP3: six-transmembrane epithelial antigen of prostate 3
HMOX1: heme oxygenase 1
IRPs: iron regulatory proteins
IREs: iron-responsive elements
HIFs: hypoxia inducible factors
TFR 1: transferrin receptor 1
TFR 2: transferrin receptor 2
JAK/STAT: Janus kinase/signal transducers and activators of transcription
MDVs: mitochondria derived vesicles
GAPDH: glyceraldehyde-3-phosphate dehydrogenase
DAP: 1,3 diaminopropane
SCFA: short chain fatty acids.

## References

[1] Singh AK. Chapter 12 – Erythropoiesis: the roles of erythropoietin and iron. 2nd ed. In: Singh AK Williams GH editors. *Textbook of Nephro-Endocrinology.* Cambridge, MA: Academic Press (2018). p. 207–15.

[2] Ward DM Cloonan SM. Mitochondrial iron in human health and disease. *Annu Rev Physiol.* (2019) 81:453–82. 10.1146/annurev-physiol-020518-114742 30485761 PMC6641538

[3] Puig S Ramos-Alonso L Romero AM Martínez-Pastor MT. The elemental role of iron in DNA synthesis and repair. *Metallomics.* (2017) 9:1483–500. 10.1039/c7mt00116a 28879348

[4] Ward RJ Crichton RR Taylor DL Corte LD Srai SK Dexter DT. Iron and the immune system. *J Neural Transmiss.* (2011) 118:315–28. 10.1007/s00702-010-0479-3 20878427

[5] Torti SV Torti FM. Iron and cancer: more ore to be mined. *Nat Rev Cancer.* (2013) 13:342–55. 10.1038/nrc3495 23594855 PMC4036554

[6] Chen X Yu C Kang R Tang D. Iron metabolism in ferroptosis. *Front Cell Dev Biol.* (2020) 8:590226. 10.3389/fcell.2020.590226 33117818 PMC7575751

[7] Ganz T Nemeth E. Hepcidin and iron homeostasis. *Biochim Biophys Acta.* (2012) 1823:1434–43. 10.1016/j.bbamcr.2012.01.014 22306005 PMC4048856

[8] Mattera VS Pereyra Gerber P Glisoni R Ostrowski M Verstraeten SV Pasquini JM Extracellular vesicles containing the transferrin receptor as nanocarriers of apotransferrin. *J Neurochem.* (2020) 155:327–38. 10.1111/jnc.15019 32248519

[9] Principe S Jones EE Kim Y Sinha A Nyalwidhe JO Brooks J In-depth proteomic analyses of exosomes isolated from expressed prostatic secretions in urine. *Proteomics.* (2013) 13:1667–71. 10.1002/pmic.201200561 23533145 PMC3773505

[10] Malhotra H Sheokand N Kumar S Chauhan AS Kumar M Jakhar P Exosomes: tunable nano vehicles for macromolecular delivery of transferrin and lactoferrin to specific intracellular compartment. *J Biomed Nanotechnol.* (2016) 12:1101–14. 10.1166/jbn.2016.2229 27305829

[11] Ruiz Martinez M Castro-Mollo M Dogra N An W Borroni E Follenzi A Role of exosomes in hepcidin regulation in β-thalassemia. *Blood.* (2019) 134(Suppl. 1):954. 10.1182/blood-2019-131094

[12] Yoon YJ Kim OY Gho YS. Extracellular vesicles as emerging intercellular communicasomes. *BMB Rep.* (2014) 47:531–9. 10.5483/bmbrep.2014.47.10.164 25104400 PMC4261509

[13] Ems T St Lucia K Huecker MR. Biochemistry iron absorption. In: *StatPearls [Internet]*. Treasure Island, FL: StatPearls Publishing (2022). 28846259

[14] Beck KL. Anemia: prevention and dietary strategies. In: Caballero B Finglas PM Toldrá F editors. *Encyclopedia of Food and Health.* Oxford: Academic Press (2016). p. 164–8.

[15] Gunshin H Fujiwara Y Custodio AO Direnzo C Robine S Andrews NC. Slc11a2 is required for intestinal iron absorption and erythropoiesis but dispensable in placenta and liver. *J Clin Invest.* (2005) 115:1258–66. 10.1172/jci24356 15849611 PMC1077176

[16] Choi J Masaratana P Latunde-Dada GO Arno M Simpson RJ McKie AT. Duodenal reductase activity and spleen iron stores are reduced and erythropoiesis is abnormal in DCYTB knockout mice exposed to hypoxic conditions. *J Nutr.* (2012) 142:1929–34. 10.3945/jn.112.160358 22990466

[17] Hentze MW Muckenthaler MU Galy B Camaschella C. Two to tango: regulation of mammalian iron metabolism. *Cell.* (2010) 142:24–38. 10.1016/j.cell.2010.06.028 20603012

[18] Kawabata H Germain RS Vuong PT Nakamaki T Said JW Koeffler HP. Transferrin receptor 2-alpha supports cell growth both in iron-chelated cultured cells and in vivo. *J Biol Chem.* (2000) 275:16618–25. 10.1074/jbc.M908846199 10748106

[19] Ohgami RS Campagna DR Greer EL Antiochos B McDonald A Chen J Identification of a ferrireductase required for efficient transferrin-dependent iron uptake in erythroid cells. *Nat Genet.* (2005) 37:1264–9. 10.1038/ng1658 16227996 PMC2156108

[20] Torti FM Torti SV. Regulation of ferritin genes and protein. *Blood.* (2002) 99:3505–16. 10.1182/blood.v99.10.3505 11986201

[21] Clara C Antonella N Laura S. Iron metabolism and iron disorders revisited in the hepcidin era. *Haematologica.* (2020) 105:260–72. 10.3324/haematol.2019.232124 31949017 PMC7012465

[22] Korolnek T Hamza I. Macrophages and iron trafficking at the birth and death of red cells. *Blood.* (2015) 125:2893–7. 10.1182/blood-2014-12-567776 25778532 PMC4424413

[23] Ward RJ Crichton RR. Chapter 15 – Iron homeostasis and aluminium toxicity. In: Exley C editor. *Aluminium and Alzheimer’s Disease.* Amsterdam: Elsevier (2001). p. 293–310.

[24] Ganz T. Systemic iron homeostasis. *Physiol Rev.* (2013) 93:1721–41. 10.1152/physrev.00008.2013 24137020

[25] Anderson CP Shen M Eisenstein RS Leibold EA. Mammalian iron metabolism and its control by iron regulatory proteins. *Biochim Biophys Acta.* (2012) 1823:1468–83. 10.1016/j.bbamcr.2012.05.010 22610083 PMC3675657

[26] Wilkinson N Pantopoulos K. The IRP/IRE system in vivo: insights from mouse models. *Front Pharmacol.* (2014) 5:176. 10.3389/fphar.2014.00176 25120486 PMC4112806

[27] Dupuy J Volbeda A Carpentier P Darnault C Moulis J-M Fontecilla-Camps JC. Crystal structure of human iron regulatory protein 1 as cytosolic aconitase. *Structure.* (2006) 14:129–39. 10.1016/j.str.2005.09.009 16407072

[28] Walden WE Selezneva AI Dupuy J Volbeda A Fontecilla-Camps JC Theil EC Structure of dual function iron regulatory protein 1 complexed with ferritin IRE-RNA. *Science.* (2006) 314:1903–8. 10.1126/science.1133116 17185597

[29] Pantopoulos K. Iron metabolism and the IRE/IRP regulatory system: an update. *Ann N Y Acad Sci.* (2004) 1012:1–13. 10.1196/annals.1306.001 15105251

[30] Andolfo I De Falco L Asci R Russo R Colucci S Gorrese M Regulation of divalent metal transporter 1 (DMT1) non-IRE isoform by the microRNA let-7d in erythroid cells. *Haematologica.* (2010) 95:1244–52. 10.3324/haematol.2009.020685 20410187 PMC2913071

[31] Schaar DG Medina DJ Moore DF Strair RK Ting Y. MIR-320 targets transferrin receptor 1 (Cd71) and inhibits cell proliferation. *Exp Hematol.* (2009) 37:245–55. 10.1016/j.exphem.2008.10.002 19135902

[32] Sangokoya C Doss JF Chi JT. Iron-responsive MIR-485-3p regulates cellular iron homeostasis by targeting ferroportin. *PLoS Genet.* (2013) 9:e1003408. 10.1371/journal.pgen.1003408 23593016 PMC3616902

[33] Shpyleva SI Tryndyak VP Kovalchuk O Starlard-Davenport A Chekhun VF Beland FA Role of ferritin alterations in human breast cancer cells. *Breast Cancer Res Treat.* (2011) 126:63–71. 10.1007/s10549-010-0849-4 20390345

[34] Shah YM Matsubara T Ito S Yim S-H Gonzalez FJ. Intestinal hypoxia-inducible transcription factors are essential for iron absorption following iron deficiency. *Cell Metab.* (2009) 9:152–64. 10.1016/j.cmet.2008.12.012 19147412 PMC2659630

[35] Mastrogiannaki M Matak P Keith B Simon MC Vaulont S Peyssonnaux C. HIF-2α, but not HIF-1α, promotes iron absorption in mice. *J Clin Invest.* (2009) 119:1159–66. 10.1172/JCI38499 19352007 PMC2673882

[36] Sanchez M Galy B Muckenthaler MU Hentze MW. Iron-regulatory proteins limit hypoxia-inducible factor-2alpha expression in iron deficiency. *Nat Struct Mol Biol.* (2007) 14:420–6. 10.1038/nsmb1222 17417656

[37] Zimmer M Ebert BL Neil C Brenner K Papaioannou I Melas A Small-molecule inhibitors of HIF-2a translation link Its 5’UTR iron-responsive element to oxygen sensing. *Mol Cell.* (2008) 32:838–48. 10.1016/j.molcel.2008.12.004 19111663 PMC3978139

[38] Anderson Sheila A Nizzi Christopher P Chang Y-I Deck Kathryn M Schmidt Paul J Galy B The IRP1-HIF-2α axis coordinates iron and oxygen sensing with erythropoiesis and iron absorption. *Cell Metab.* (2013) 17:282–90. 10.1016/j.cmet.2013.01.007 23395174 PMC3612289

[39] Maria M Pavle M Jacques RRM Stéphanie D Patrick M Sophie V Hepatic hypoxia-inducible factor-2 down-regulates hepcidin expression in mice through an erythropoietin-mediated increase in erythropoiesis. *Haematologica.* (2012) 97:827–34. 10.3324/haematol.2011.056119 22207682 PMC3366646

[40] Liu Q Davidoff O Niss K Haase VH. Hypoxia-inducible factor regulates hepcidin via erythropoietin-induced erythropoiesis. *J Clin Invest.* (2012) 122:4635–44. 10.1172/JCI63924 23114598 PMC3533545

[41] Parrow NL Fleming RE. Bone morphogenetic proteins as regulators of iron metabolism. *Annu Rev Nutr.* (2014) 34:77–94. 10.1146/annurev-nutr-071813-105646 24995692 PMC7713507

[42] Camaschella C Pagani A Nai A Silvestri L. The mutual control of iron and erythropoiesis. *Int J Lab Hematol.* (2016) 38:20–6. 27161430 10.1111/ijlh.12505

[43] Forejtnikova H Vieillevoye M Zermati Y Lambert M Pellegrino RM Guihard S Transferrin receptor 2 is a component of the erythropoietin receptor complex and is required for efficient erythropoiesis. *Blood.* (2010) 116:5357–67. 10.1182/blood-2010-04-281360 20826723

[44] Wrighting DM Andrews NC. Interleukin-6 induces hepcidin expression through STAT3. *Blood.* (2006) 108:3204–9. 10.1182/blood-2006-06-027631 16835372 PMC1895528

[45] Nemeth E Tuttle MS Powelson J Vaughn MB Donovan A Ward DM Hepcidin regulates cellular iron efflux by binding to ferroportin and inducing its internalization. *Science.* (2004) 306:2090–3. 10.1126/science.1104742 15514116

[46] Doyle LM Wang MZ. Overview of extracellular vesicles, their origin, composition, purpose, and methods for exosome isolation and analysis. *Cells.* (2019) 8:727. 10.3390/cells8070727 31311206 PMC6678302

[47] Bang C Thum T. Exosomes: new players in cell-cell communication. *Int J Biochem Cell Biol.* (2012) 44:2060–4. 10.1016/j.biocel.2012.08.007 22903023

[48] Rouault TA Tang CK Kaptain S Burgess WH Haile DJ Samaniego F Cloning of the CDNA encoding an RNA regulatory protein–the human iron-responsive element-binding protein. *Proc Natl Acad Sci U.S.A.* (1990) 87:7958–62. 10.1073/pnas.87.20.7958 2172968 PMC54871

[49] Mancias JD Wang X Gygi SP Harper JW Kimmelman AC. Quantitative proteomics identifies NCOA4 as the cargo receptor mediating ferritinophagy. *Nature.* (2014) 509:105–9. 10.1038/nature13148 24695223 PMC4180099

[50] Mancias JD Pontano Vaites L Nissim S Biancur DE Kim AJ Wang X Ferritinophagy via NCOA4 is required for erythropoiesis and is regulated by iron dependent HERC2-mediated proteolysis. *Elife.* (2015) 4:e10308. 10.7554/eLife.10308 26436293 PMC4592949

[51] Harrison PM Arosio P. The ferritins: molecular properties, iron storage function and cellular regulation. *Biochim Biophys Acta.* (1996) 1275:161–203. 10.1016/0005-272800022-9 8695634

[52] Cai CX Birk DE Linsenmayer TF. Nuclear ferritin protects DNA from UV damage in corneal epithelial cells. *Mol Biol Cell.* (1998) 9:1037–51. 10.1091/mbc.9.5.1037 9571238 PMC25328

[53] Levi S Corsi B Bosisio M Invernizzi R Volz A Sanford D A human mitochondrial ferritin encoded by an intronless gene. *J Biol Chem.* (2001) 276:24437–40. 11323407 10.1074/jbc.C100141200

[54] Ghosh S Hevi S Chuck SL. Regulated secretion of glycosylated human ferritin from hepatocytes. *Blood.* (2004) 103:2369–76. 10.1182/blood-2003-09-3050 14615366

[55] Cohen LA Gutierrez L Weiss A Leichtmann-Bardoogo Y Zhang DL Crooks DR Serum ferritin is derived primarily from macrophages through a nonclassical secretory pathway. *Blood.* (2010) 116:1574–84. 10.1182/blood-2009-11-253815 20472835

[56] Truman-Rosentsvit M Berenbaum D Spektor L Cohen LA Belizowsky-Moshe S Lifshitz L Ferritin is secreted via 2 distinct nonclassical vesicular pathways. *Blood.* (2018) 131:342–52. 10.1182/blood-2017-02-768580 29074498 PMC5774206

[57] Yanatori I Richardson DR Dhekne HS Toyokuni S Kishi F. Cd63 is regulated by iron via the IRE-IRP system and is important for ferritin secretion by extracellular vesicles. *Blood.* (2021) 138:1490–503. 10.1182/blood.2021010995 34265052 PMC8667049

[58] Hurwitz SN Conlon MM Rider MA Brownstein NC Meckes DG Jr. Nanoparticle analysis sheds budding insights into genetic drivers of extracellular vesicle biogenesis. *J Extracell Vesicles.* (2016) 5:31295. 10.3402/jev.v5.31295 27421995 PMC4947197

[59] Eisenstein RS Blemings KP. Iron regulatory proteins, iron responsive elements and iron homeostasis. *J Nutr.* (1998) 128:2295–8. 10.1093/jn/128.12.2295 9868172

[60] Vasam G Nadeau R Cadete VJJ Lavallée-Adam M Menzies KJ Burelle Y. Proteomics characterization of mitochondrial-derived vesicles under oxidative stress. *FASEB J.* (2021) 35:e21278. 10.1096/fj.202002151R 33769614 PMC8252493

[61] Daru J Colman K Stanworth SJ De La Salle B Wood EM Pasricha S-R. Serum ferritin as an indicator of iron status: what do we need to know? *Am J Clin Nutr.* (2017) 106(Suppl. 6):1634S–9S. 10.3945/ajcn.117.155960 29070560 PMC5701723

[62] Thiengtavor C Siriworadetkun S Paiboonsukwong K Fucharoen S Pattanapanyasat K Vadolas J Increased ferritin levels in non-transfusion-dependent β^°^-thalassaemia/HBE are associated with reduced CXCR2 expression and neutrophil migration. *Br J Haematol.* (2020) 189:187–98. 10.1111/bjh.16295 31884679

[63] Pootrakul P Vongsmasa V La-ongpanich P Wasi P. Serum ferritin levels in thalassemias and the effect of splenectomy. *Acta Haematol.* (1981) 66:244–50. 10.1159/000207129 6800190

[64] Kittivorapart J Crew VK Wilson MC Heesom KJ Siritanaratkul N Toye AM. Quantitative proteomics of plasma vesicles identify novel biomarkers for hemoglobin E/β-thalassemic patients. *Blood Adv.* (2018) 2:95–104. 10.1182/bloodadvances.2017011726 29365317 PMC5787864

[65] Atipimonpat A Siwaponanan P Khuhapinant A Svasti S Sukapirom K Khowawisetsut L Extracellular vesicles from thalassemia patients carry iron-containing ferritin and hemichrome that promote cardiac cell proliferation. *Ann Hematol.* (2021) 100:1929–46. 10.1007/s00277-021-04567-z 34155536

[66] Strzyz P. Iron expulsion by exosomes drives ferroptosis resistance. *Nat Rev Mol Cell Biol.* (2020) 21:4–5. 10.1038/s41580-019-0195-2 31748716

[67] Mukherjee C Kling T Russo B Miebach K Kess E Schifferer M Oligodendrocytes provide antioxidant defense function for neurons by secreting ferritin heavy chain. *Cell Metab.* (2020) 32:259–72.e10. 10.1016/j.cmet.2020.05.019 32531201 PMC7116799

[68] Brown CW Mercurio AM. Ferroptosis resistance mediated by exosomal release of iron. *Mol Cell Oncol.* (2020) 7:1730144. 10.1080/23723556.2020.1730144 32391424 PMC7199748

[69] Ito F Kato K Yanatori I Murohara T Toyokuni S. Ferroptosis-dependent extracellular vesicles from macrophage contribute to asbestos-induced mesothelial carcinogenesis through loading ferritin. *Redox Biol.* (2021) 47:102174. 10.1016/j.redox.2021.102174 34700146 PMC8577498

[70] Osti D Del Bene M Rappa G Santos M Matafora V Richichi C Clinical significance of extracellular vesicles in plasma from glioblastoma patients. *Clin Cancer Res.* (2019) 25:266–76. 10.1158/1078-0432.ccr-18-1941 30287549

[71] Kumar S Sheokand N Mhadeshwar MA Raje CI Raje M. Characterization of glyceraldehyde-3-phosphate dehydrogenase as a novel transferrin receptor. *Int J Biochem Cell Biol.* (2012) 44:189–99. 10.1016/j.biocel.2011.10.016 22062951

[72] Sheokand N Kumar S Malhotra H Tillu V Raje CI Raje M. Secreted glyceraldehye-3-phosphate dehydrogenase is a multifunctional autocrine transferrin receptor for cellular iron acquisition. *Biochim Biophys Acta.* (2013) 1830:3816–27. 10.1016/j.bbagen.2013.03.019 23541988

[73] Dar GH Mendes CC Kuan W-L Speciale AA Conceição M Görgens A GAPDH controls extracellular vesicle biogenesis and enhances the therapeutic potential of EV mediated siRNA delivery to the brain. *Nat Commun.* (2021) 12:6666. 10.1038/s41467-021-27056-3 34795295 PMC8602309

[74] Liu X-B Nguyen N-BH Marquess KD Yang F Haile DJ. Regulation of hepcidin and ferroportin expression by lipopolysaccharide in splenic macrophages. *Blood Cells Mol Dis.* (2005) 35:47–56. 10.1016/j.bcmd.2005.04.006 15932798

[75] Bekri S Gual P Anty R Luciani N Dahman M Ramesh B Increased adipose tissue expression of hepcidin in severe obesity is independent from diabetes and nash. *Gastroenterology.* (2006) 131:788–96. 10.1053/j.gastro.2006.07.007 16952548

[76] Bessman NJ Mathieu JRR Renassia C Zhou L Fung TC Fernandez KC Dendritic cell-derived hepcidin sequesters iron from the microbiota to promote mucosal healing. *Science.* (2020) 368:186–9. 10.1126/science.aau6481 32273468 PMC7724573

[77] Zhang V Nemeth E Kim A. Iron in lung pathology. *Pharmaceuticals.* (2019) 12:30. 10.3390/ph12010030 30781366 PMC6469192

[78] Kulaksiz H Theilig F Bachmann S Gehrke SG Rost D Janetzko A The iron-regulatory peptide hormone hepcidin: expression and cellular localization in the mammalian kidney. *J Endocrinol.* (2005) 184:361–70. 10.1677/joe.1.05729 15684344

[79] Schwarz P Kübler JA Strnad P Müller K Barth TF Gerloff A Hepcidin is localised in gastric parietal cells, regulates acid secretion and is induced by *Helicobacter pylori* infection. *Gut.* (2012) 61:193–201. 10.1136/gut.2011.241208 21757452

[80] Zechel S Huber-Wittmer K von Bohlen und Halbach O. Distribution of the iron-regulating protein hepcidin in the murine central nervous system. *J Neurosci Res.* (2006) 84:790–800. 10.1002/jnr.20991 16933319

[81] Merle U Fein E Gehrke SG Stremmel W Kulaksiz H. The iron regulatory peptide hepcidin is expressed in the heart and regulated by hypoxia and inflammation. *Endocrinology.* (2007) 148:2663–8. 10.1210/en.2006-1331 17363462

[82] Malerba M Louis S Cuvellier S Shambat SM Hua C Gomart C Epidermal hepcidin is required for neutrophil response to bacterial infection. *J Clin Invest.* (2020) 130:329–34. 10.1172/JCI126645 31600168 PMC6934188

[83] Lakhal-Littleton S Wolna M Chung YJ Christian HC Heather LC Brescia M An essential cell-autonomous role for hepcidin in cardiac iron homeostasis. *Elife.* (2016) 5:e19804. 10.7554/eLife.19804 27897970 PMC5176354

[84] Arezes J Foy N McHugh K Sawant A Quinkert D Terraube V Erythroferrone inhibits the induction of hepcidin by BMP6. *Blood.* (2018) 132:1473–7. 10.1182/blood-2018-06-857995 30097509 PMC6238155

[85] Gardenghi S Grady RW Rivella S. Anemia, ineffective erythropoiesis, and hepcidin: interacting factors in abnormal iron metabolism leading to iron overload in β-thalassemia. *Hematol Oncol Clin North Am.* (2010) 24:1089–107. 10.1016/j.hoc.2010.08.003 21075282 PMC2991049

[86] Levin C Koren A Rebibo-Sabbah A Levin M Koifman N Brenner B Extracellular vesicle microrna that are involved in β-thalassemia complications. *Int J Mol Sci.* (2021) 22:9760. 10.3390/ijms22189760 34575936 PMC8465435

[87] Sharp P Srai SK. Molecular mechanisms involved in intestinal iron absorption. *World J Gastroenterol.* (2007) 13:4716–24. 10.3748/wjg.v13.i35.4716 17729393 PMC4611193

[88] Donaldson GP Lee SM Mazmanian SK. Gut biogeography of the bacterial microbiota. *Nat Rev Microbiol.* (2016) 14:20–32. 10.1038/nrmicro3552 26499895 PMC4837114

[89] Bäckhed F Ley RE Sonnenburg JL Peterson DA Gordon JI. Host-bacterial mutualism in the human intestine. *Science.* (2005) 307:1915–20. 10.1126/science.1104816 15790844

[90] Dostal A Chassard C Hilty FM Zimmermann MB Jaeggi T Rossi S Iron depletion and repletion with ferrous sulfate or electrolytic iron modifies the composition and metabolic activity of the gut microbiota in rats. *J Nutr.* (2011) 142:271–7. 10.3945/jn.111.148643 22190022 PMC3260059

[91] Buhnik-Rosenblau K Moshe-Belizowski S Danin-Poleg Y Meyron-Holtz EG. Genetic modification of iron metabolism in mice affects the gut microbiota. *Biometals.* (2012) 25:883–92. 10.1007/s10534-012-9555-5 22580926

[92] Liu B Pan X Liu Z Han M Xu G Dai X Fecal microbiota as a noninvasive biomarker to predict the tissue iron accumulation in intestine epithelial cells and liver. *FASEB J.* (2020) 34:3006–20. 10.1096/fj.201901635RR 31912587

[93] Deschemin J-C Noordine M-L Remot A Willemetz A Afif C Canonne-Hergaux F The microbiota shifts the iron sensing of intestinal cells. *FASEB J.* (2015) 30:252–61. 10.1096/fj.15-276840 26370847

[94] Das NK Schwartz AJ Barthel G Inohara N Liu Q Sankar A Microbial metabolite signaling is required for systemic iron homeostasis. *Cell Metab.* (2020) 31:115–30.e6. 10.1016/j.cmet.2019.10.005 31708445 PMC6949377

[95] Taylor M Qu A Anderson ER Matsubara T Martin A Gonzalez FJ Hypoxia-inducible factor-2α mediates the adaptive increase of intestinal ferroportin during iron deficiency in mice. *Gastroenterology.* (2011) 140:2044–55. 10.1053/j.gastro.2011.03.007 21419768 PMC3109109

[96] Zhang D Gao X Li H Borger DK Wei Q Yang E The microbiota regulates hematopoietic stem cell fate decisions by controlling iron availability in bone marrow. *Cell Stem Cell.* (2022) 29:232–47.e7. 10.1016/j.stem.2021.12.009 35065706 PMC8818037

[97] Nicolas G Chauvet C Viatte L Danan JL Bigard X Devaux I The gene encoding the iron regulatory peptide hepcidin is regulated by anemia, hypoxia, and inflammation. *J Clin Invest.* (2002) 110:1037–44. 10.1172/JCI15686 12370282 PMC151151

[98] Aschemeyer S Qiao B Stefanova D Valore EV Sek AC Ruwe TA Structure-function analysis of ferroportin defines the binding site and an alternative mechanism of action of hepcidin. *Blood.* (2018) 131:899–910. 10.1182/blood-2017-05-786590 29237594 PMC5824336

[99] Clarke G Stilling RM Kennedy PJ Stanton C Cryan JF Dinan TG. Minireview: gut microbiota: the neglected endocrine organ. *Mol Endocrinol.* (2014) 28:1221–38. 10.1210/me.2014-1108 24892638 PMC5414803

[100] Peyssonnaux C Zinkernagel AS Datta V Lauth X Johnson RS Nizet V. TLR4-dependent hepcidin expression by myeloid cells in response to bacterial pathogens. *Blood.* (2006) 107:3727–32. 10.1182/blood-2005-06-2259 16391018 PMC1895778

[101] Layoun A Santos MM. Bacterial cell wall constituents induce hepcidin expression in macrophages through MYD88 signaling. *Inflammation.* (2012) 35:1500–6. 10.1007/s10753-012-9463-4 22544439

[102] Seyoum Y Baye K Humblot C. Iron homeostasis in host and gut bacteria – A complex interrelationship. *Gut Microbes.* (2021) 13:1874855. 10.1080/19490976.2021.1874855 33541211 PMC7872071

[103] Jan AT. Outer membrane vesicles (OMVS) of gram-negative bacteria: a perspective update. *Front Microbiol.* (2017) 8:1053. 10.3389/fmicb.2017.01053 28649237 PMC5465292

[104] Bose S Aggarwal S Singh DV Acharya N. Extracellular vesicles: an emerging platform in gram-positive bacteria. *Microbial Cell.* (2020) 7:312–22. 10.15698/mic2020.12.737 33335921 PMC7713254

[105] Prados-Rosales R Weinrick BC Piqué DG Jacobs WR Casadevall A Rodriguez GM. Role for *Mycobacterium tuberculosis* membrane vesicles in iron acquisition. *J Bacteriol.* (2014) 196:1250–6. 10.1128/JB.01090-13 24415729 PMC3957709

[106] Wang M Nie Y Wu X-L. Membrane vesicles from a *Dietzia* bacterium containing multiple cargoes and their roles in iron delivery. *Environ Microbiol.* (2021) 23:1009–19. 10.1111/1462-2920.15278 33048442

[107] Ñahui Palomino RA Vanpouille C Costantini PE Margolis L. Microbiota–host communications: bacterial extracellular vesicles as a common language. *PLoS Pathog.* (2021) 17:e1009508. 10.1371/journal.ppat.1009508 33984071 PMC8118305

[108] Díaz-Garrido N Badia J Baldomà L. Microbiota-derived extracellular vesicles in interkingdom communication in the gut. *J Extracell Vesicles.* (2021) 10:e12161. 10.1002/jev2.12161 34738337 PMC8568775

[109] Díez-Sainz E Milagro FI Riezu-Boj JI Lorente-Cebrián S. Effects of gut microbiota–derived extracellular vesicles on obesity and diabetes and their potential modulation through diet. *J Physiol Biochem.* (2022) 78:485–99. 10.1007/s13105-021-00837-6 34472032 PMC8410452

[110] Smith JL. The physiological role of ferritin-like compounds in bacteria. *Crit Rev Microbiol.* (2004) 30:173–85. 10.1080/10408410490435151 15490969

[111] Arosio P Elia L Poli M. Ferritin, cellular iron storage and regulation. *IUBMB Life.* (2017) 69:414–22. 10.1002/iub.1621 28349628

[112] Zakharzhevskaya NB Vanyushkina AA Altukhov IA Shavarda AL Butenko IO Rakitina DV Outer membrane vesicles secreted by pathogenic and nonpathogenic *Bacteroides fragilis* represent different metabolic activities. *Sci Rep.* (2017) 7:5008. 10.1038/s41598-017-05264-6 28694488 PMC5503946

[113] Hu R Lin H Wang M Zhao Y Liu H Min Y *Lactobacillus reuteri*-derived extracellular vesicles maintain intestinal immune homeostasis against lipopolysaccharide-induced inflammatory responses in broilers. *J Anim Sci Biotechnol.* (2021) 12:25. 10.1186/s40104-020-00532-4 33593426 PMC7888134

[114] Dean SN Rimmer MA Turner KB Phillips DA Caruana JC Hervey WJT *Lactobacillus acidophilus* membrane vesicles as a vehicle of bacteriocin delivery. *Front Microbiol.* (2020) 11:710. 10.3389/fmicb.2020.00710 32425905 PMC7203471

[115] Olaya-Abril A Prados-Rosales R McConnell MJ Martín-Peña R González-Reyes JA Jiménez-Munguía I Characterization of protective extracellular membrane-derived vesicles produced by *Streptococcus pneumoniae*. *J Proteomics.* (2014) 106:46–60. 10.1016/j.jprot.2014.04.023 24769240

[116] Matsumoto J Stewart T Banks WA Zhang J. The transport mechanism of extracellular vesicles at the blood-brain barrier. *Curr Pharmaceut Design.* (2017) 23:6206–14. 10.2174/1381612823666170913164738 28914201

[117] Gebrayel P Nicco C Al Khodor S Bilinski J Caselli E Comelli EM Microbiota medicine: towards clinical revolution. *J Transl Med.* (2022) 20:111. 10.1186/s12967-022-03296-9 35255932 PMC8900094

